# Forecasting Ethiopia's Neonatal Mortality Rate Toward the Sustainable Development Goal Target 3.2 Using Statistical and Deep Learning Models: A Time‐Series Forecasting Study

**DOI:** 10.1002/hsr2.73402

**Published:** 2026-10-21

**Authors:** Fentahun Bikale Kebede, Selam Kifle Dergba, Amanuel Worku

**Affiliations:** ^1^ Strategic Affairs Executive Office Ministry of Health Addis Ababa Ethiopia; ^2^ College of Computational and Natural Science Addis Ababa University Addis Ababa Ethiopia

**Keywords:** ARIMA, long short‐term memory, neonatal mortality rate, time series forecasting

## Abstract

**Background and Aims:**

A high neonatal mortality rate (NMR) reflects underlying health inequalities, the quality of maternal and newborn care, and overall healthcare system performance. Despite substantial progress, Ethiopia remains off track to achieve Sustainable Development Goal (SDG) 3.2, which aims to reduce NMR to at least 12/1000 by 2030. This study compared statistical and deep learning models to forecast Ethiopia's NMR through 2030, assess the progress towards the SDG target, and provide evidence to inform policy.

**Methods:**

Annual NMR data were obtained from the WHO Health Inequality Data Repository. Autoregressive integrated moving average (ARIMA), long short‐term memory (LSTM), multi‐step LSTM, and a hybrid were developed using Python. For the deep learning models, the data were normalised using MinMax scaling and transformed into sequential time‐series windows. Stationarity was assessed using rolling statistics and the Augmented Dickey–Fuller test, while ARIMA parameters were selected using autocorrelation and partial autocorrelation and diagnostic criteria. Model performance was evaluated on the test dataset using mean absolute error (MAE), root mean square error (RMSE), mean absolute percentage error (MAPE), symmetric MAPE (sMAPE), and mean absolute scaled error (MASE). The best‐performing model was used to forecast NMR from 2024 to 2030.

**Results:**

Ethiopia's NMR decreased from 66.39 to 27.39 deaths per 1000 live births between 1977 and 2023. ARIMA offers the best performance, with MAE of 0.10, RMSE of 0.12, MAPE of 0.32, sMAPE of 0.32, and MASE of 0.11. The forecast indicates a gradual decline in Ethiopia's NMR, with the ARIMA model forecasting an NMR of 22.72 per 1000 live births (95% PI: 17.60–27.84) by 2030.

**Conclusion:**

Ethiopia's decline in NMR is insufficient to meet SDG target 3.2 by 2030. The ARIMA model's strong performance confirms traditional methods suit short‐term forecasts. To further reduce NMR and advance the SDGs, accelerated implementation of evidence‐based interventions, strengthened health systems, and ongoing monitoring are crucial.

**Abbreviations**
ADF: Augmented Dickey–Fuller
ARIMA: autoregressive integrated moving average
GBD: global burden of disease
LSTM: long short‐term memory
MAE: mean absolute error
MAPE: mean absolute percentage error
NMR: neonatal mortality rate
PACF: partial autocorrelation function
RMSE: root mean square error
RNN: recurrent neural network
SDG: sustainable development goals
sMAPE: symmetric mean absolute percentage error
WHO: World Health Organisation

## 1 Background

A high neonatal mortality rate in a country reflects underlying inequities and the quality of maternal newborn care, and it serves as a highly sensitive indicator of the strength of the healthcare system [1, 2, 3]. According to the report of the WHO in 2022, globally, 2.3 million newborns died in 2022, and premature death, birth complications, neonatal infection, and congenital problems remain the leading causes of neonatal deaths [4, 5]. This accounts for almost half (47%) of the deaths that occurred in those under 5 years of age. Sub‐Saharan Africa has the highest NMR, with 27 deaths per 1000 live births; subsequently, Central and Southern Asia follow with an NMR of 21 deaths per 1000 live births [5]. Providing effective strategies and interventions to those who need them can improve the NMR; however, this remains a challenge in the sub‐Saharan region [6].

Globally, NMR has shown significant improvement, although it now accounts for 47% of all under‐5 deaths, up from 40% in 1990, because deaths among older children are declining more rapidly [4]. In Africa, specifically in sub‐Saharan Africa, there has been notable but slow progress in NMR, which remains the region with the highest burden of neonatal death. Many African countries, particularly those in sub‐Saharan Africa, are off track to achieve the United Nations SDG Target 3.2, which aims to reduce the NMR to at least 12/1000 live births by 2030 [1].

According to the 2019 Ethiopian Mini Demographic Health Survey (EMDHS) report, the NMR was 33 deaths per 1000 live births, with regional variation ranging from 17 per 1000 live births in Addis Ababa to 55 per 1000 live births in the Benishangul–Gumuz region [7, 8]. This figure shows an increase from the 2016 EDHS report, which was 29 per 1000 live births [8]. Different studies identified different factors associated with this increased NMR, such as low maternal and neonatal healthcare utilisation [9], maternal and husband's education [10], wealth status and residency, preterm births, low birth weight, multiple births [11], adolescent (< 18) and older (> 35) mothers [9], regional disparities, and delayed breastfeeding practice [9, 11].

In line with the SDGs, Ethiopia has developed its Health Sector Transformation Plan II [7], which was later amended to the Health Sector Medium‐term Development and Investment Plan (2023–2025) [8], aimed at reducing the NMR to 21 deaths per 1000 live births by 2025. Due to the expansion of health extension programmes [9], community‐based newborn care, an increase in skilled birth attendance [10], and the integrated management of neonatal and childhood illnesses [11], Ethiopia has achieved a remarkable reduction in NMR over the past two decades [12]. However, in recent years, the rate of decline has slowed compared to earlier periods, requiring an accelerated reduction through a comprehensive, equity‐focused, and quality‐centred approach integrated with community participation [11].

Nowadays, the rapid advancement in artificial intelligence has led to the widespread utilisation of statistical methods for modelling and prediction. Some studies have used deep learning and machine learning to predict child health indicators [13, 14, 15, 16, 17]. ARIMA and LSTM are widely used for analysing and evaluating mortality time series of children, including NMR [18].

The ARIMA model is valuable for analysing non‐stationary time series data, with its main strength being flexibility, as it can be applied to a wide range of time series datasets [19]. Additionally, it can effectively capture complex variations, particularly during periods of rapid data change. Traditionally, time series analysis has relied on parametric models shaped by domain expertise, such as autoregressive models and exponential smoothing techniques [20]. However, modern machine learning models offer alternatives by enabling the modelling of temporal patterns solely through data‐driven learning [17].

LSTMs were introduced to address the limitations of recurrent neural Networks (RNNs), which faced challenges in capturing long‐term dependencies in sequential data [21]. The concepts of gating mechanisms (forget gate, input gate, and output gate) were introduced by LSTM to regulate the flow of information. However, two key problems were identified that can hinder the performance of machine learning models [22]. The first is that their high flexibility may lead to overfitting, especially on small datasets. The second point is that machine learning models are highly dependent on proper data preprocessing, which significantly impacts their predictive accuracy [22].

The development of hybrid models is growing to overcome the limitations of traditional models and machine learning models. These hybrid models showed improved performance across different scenarios. Distinguishing between stationary and non‐stationary components of the time series is a primary advantage, as it reduces the need for extensive input preprocessing [23]. The ARIMA + LSTM hybrid model has been adopted to leverage the strengths of both linear and nonlinear models, combining ARIMA's capability to model linearity with LSTM's strength in capturing nonlinear dynamics, resulting in more accurate short‐term predictions [23]. Moreover, multi‐step LSTM is designed to generate predictions across multiple future points [24].

Therefore, this study aimed to forecast Ethiopia's NMR from 2024 to 2030 by comparing the forecasting performance of ARIMA, LSTM, multi‐step LSTM, and hybrid ARIMA‐LSTM models. In addition, the study assessed Ethiopia's projected progress toward achieving SDG target 3.2 and provided evidence to support future maternal and newborn health policy, strategic planning, and resource prioritisation.

## 2 Methods

### 2.1 Data Source and Setting

Neonatal and under‐5 mortality estimates of Ethiopia from 1977 to 2023 were obtained from the WHO Health Inequality Data Repository (https://www.who.int/data/inequality-monitor). The Health Inequality Data Repository was launched in 2023 and is the largest publicly available, open, and disaggregated dataset on health and its determinants. The database has more than 16 data sources, 2000 indicators, including reproductive, maternal, newborn, child health indicators, and diverse dimensions of inequalities such as Age, Birth order, Disability, Education, economic status, Employment status, Marital Status, place of residence, number of living children, religion, subnational region, and child's sex from multicounty household surveys which are comparable across the countries and over time from 1950 to 2023. Detailed estimates of maternal and child health and other health indicators are found in this publication [25]. The NMR per 1000 live births data for both sexes in Ethiopia were extracted annually from 1977 to 2023 at https://www.who.int/data/inequality-monitor/data.

Before analysis, the dataset was inspected for completeness, temporal continuity, duplication, and consistency of measurements across years. No missing data were found.

### 2.2 Operational Definition

WHO defines NMR as the number of deaths during the first 28 completed days of life in a given year or other period. It can be subclassified as early neonatal deaths occurring in the first 7 days of life, and late neonatal deaths that occur after the seventh day but before the 28th completed day of life [26].

### 2.3 Data Preprocessing

Integrated approaches combining statistical models and deep learning algorithms were employed to improve forecasting accuracy. Before model development, the dataset underwent structured preprocessing. Firstly, the annual estimates were ordered chronologically to preserve the temporal dependency inherent in time series data. For statistical modelling, the original NMR time series was analysed directly. Stationarity was assessed using rolling mean and rolling standard deviation plots followed by formal statistical testing using the Augmented Dickey–Fuller (ADF) test. Since the original series remained non‐stationary (ADF statistic = −2.02, *p* = 0.28), successive differencing was employed. First‐order differencing resulted in a stationary series, with an ADF statistic of −4.5 and *p* < 0.001. Consequently, the differencing order was selected based on empirical evidence. Based on the seasonal decomposition, no meaningful seasonality was detected; therefore, seasonal ARIMA models were not considered.

For deep learning models, the data were normalised using the MinMax function from scikit‐learn, which maps values to the range 0–1 to facilitate efficient neural network optimisation. The data were then organised as independent variables (X) and target variables (y). Finally, this study employed four models: ARIMA, a statistical time series model; LSTM; Multistep LSTM; and Hybrid Model (ARIMA + LSTM). All statistical analyses were conducted using Python version 3.14.5. Data manipulation was done with pandas and numpy. Time series analysis was performed using the statsmodels library. Deep learning models were developed using TensorFlow 2.20.0 with the Keras 3.11.3 API. Data visualisation was performed using Matplotlib.

### 2.4 Training and Testing Strategy

To preserve the chronological structure and prevent information leakage, a sequential train‐test split was done with an 80/20 ratio. After identifying the high‐performing time series model, the selected model was refitted using the historical dataset (1977–2023) to generate forecasts for the period 2024–2030.

### 2.5 ARIMA Model Development

The ARIMA model was developed following Box–Jenkins methodology, which consists of model identification, parameter estimation, diagnostic evaluation, and forecasting. ARIMA is a statistical model, where ‘AR’ represents autoregression, which shows the relationship between current values and the present values with the assumption of the past values influence the present values, ‘I’ means the number of times the data differenced to become stationary by removing trends or seasonality, and ‘MA’ stands for moving average, which is the sum of error terms or residuals in autoregression [19]. ARIMA is represented as ARIMA (*p, d, q*), where *p* is the order of the autoregression, *d* is the order of differencing, and *q* is the order of error terms or moving average. Following confirmation of stationarity, candidate *p* and *q* orders were identified by examining the autocorrelation (ACF) and partial autocorrelation (PACF) plots (Figure 1). Multiple ARIMA models were subsequently fitted and compared on the validation dataset using the Akaike Information Criterion (AIC), Bayesian Information Criterion (BIC), and root mean squared error (RMSE). The final model was selected based on the combination of Lower AIC, BIC, and RMSE. Model accuracy was further examined using residual plots, histograms, quantile–quantile plots, and residual autocorrelation. The Ljung–Box test was performed, and the non‐significant result indicates that the selected model adequately captured the temporal dependence in the observed series.

**Figure 1:**
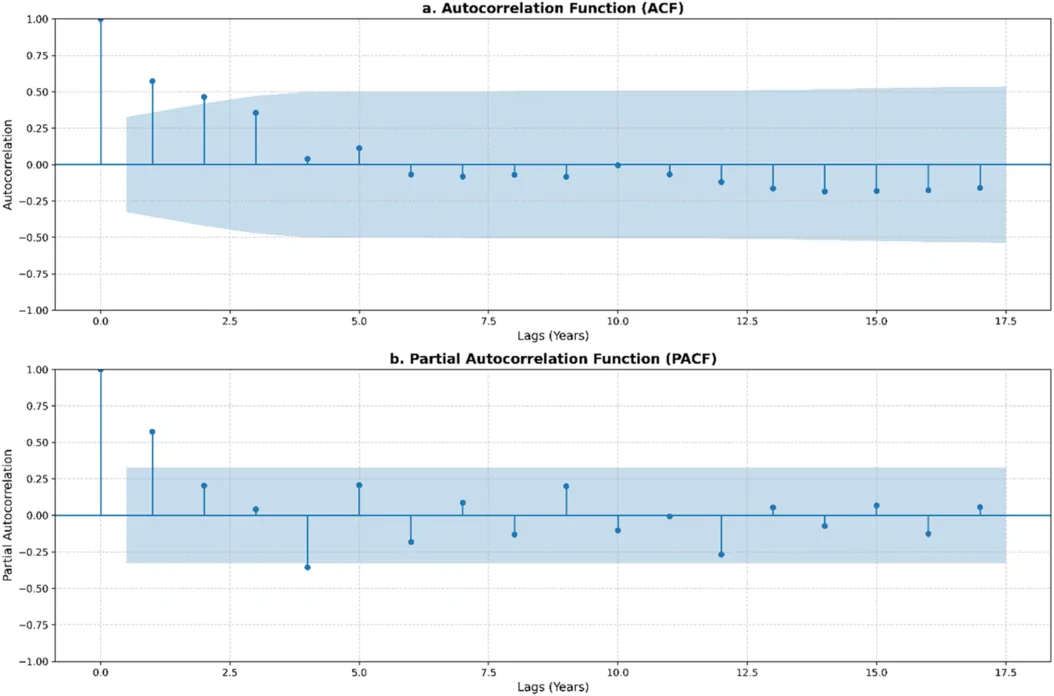
Autocorrelation (a) and partial autocorrelation function (b) plots of the differenced neonatal mortality time series in Ethiopia.

### 2.6 Deep Learning and Hybrid Model Development

Long short‐term memory (LSTM) is an extension of a recurrent neural network that utilises memory cells and gates to regulate the flow of information, enabling the network to efficiently retain or discard data over extended sequences. Therefore, LSTM contains units called memory blocks in the hidden layer, which might include one or more memory cells, and have input and output ports to control the flow of information. The input gate regulates the flow of input activation, and the output gate regulates the flow of output activation. Later, a ‘forget gate’ was added to the memory blocks [27].

The network architecture consisted of a single LSTM hidden layer containing 50 memory units, followed by a fully connected dense output layer. Adam optimiser with a learning rate of 0.001 and a batch size of 32 was used for a maximum of epochs. An early stopping callback was implemented to monitor validation loss and terminate training after a predefined number of epochs with no further improvement, while restoring the optimal model weights, thereby minimising overfitting.

For the multi‐step LSTM, the same preprocessing procedures, normalisation strategy, and sliding window transformation were applied.

Furthermore, the hybrid (ARIMA + LSTM) model was developed by combining ARIMA, which was first designed to capture linear components, with LSTM, which captures residual errors (nonlinear patterns), thereby leveraging the strengths of both models. This combination aims to enhance forecasting accuracy by augmenting the statistical power of ARIMA with the feature extraction capabilities of LSTM.

### 2.7 Model Evaluation and Future Forecasting

The evaluation of the models was conducted using standard error metrics, including Root Mean Square Error (RMSE), Mean Absolute Error (MAE), Mean Absolute Percentage Error (MAPE), and symmetric mean absolute percentage error (sMAPE) calculated as (Equations 1 to 5).

(1) $$\mathrm{RMSE}=\sqrt{(\frac{1}{n}\sum_{i=1}^{n}(}{\hat{y}-\mathrm{yi})}^{2}$$

(2) $$\mathrm{MAE}=\frac{1}{n}\;\sum_{i=1}^{n}|\hat{y}-y_{i}|$$

(3) $$\mathrm{MAPE}=\frac{1}{n}\sum_{i=1}^{n}\frac{\left|\hat{y}-y_{i}\right|}{y_{i}}*100$$

(4) $$\mathrm{sMAPE}=\;\frac{100\%}{n}\sum_{i=1}^{n}\frac{2|\hat{y}-y_{i}|}{\hat{|y}|+|y_{i}|}$$

(5) $$\mathrm{MASE}=\frac{\frac{1}{n}\sum_{i=1}^{n}|{\hat{y}}_{i}-y_{i}|}{\frac{1}{n-m}\sum_{t=m+1}^{n}{|y_{t}-y}_{t-m}|}$$

where $\hat{y}$ is the forecasted value, $y_{i}$ is the observed value, *m* is annual seasonality, and *n* is the length of the series.

Forecasts were applied for the period 2024–2030, with 95% prediction intervals (I) calculated to quantify forecast uncertainty and provide a plausible range for future NMR.

### 2.8 Ethical Statement

This study used publicly available, de‐identified secondary data from the WHO Health Inequality Monitor database. Ethical approval and informed consent were not required because the data are publicly available and contain no personally identifiable information.

## 3 Results

### 3.1 Temporal Trend of Neonatal Mortality in Ethiopia

Ethiopia has reduced the overall NMR from 66.39 to 27.39 between 1977 and 2023, as reported in the UN IGME and the WHO report. The overall reduction was approximately 58.7%. Despite this substantial reduction, the NMR remained much higher than the SDG target of 12 deaths per 1000 live births by 2030 (Figure 2).

**Figure 2:**
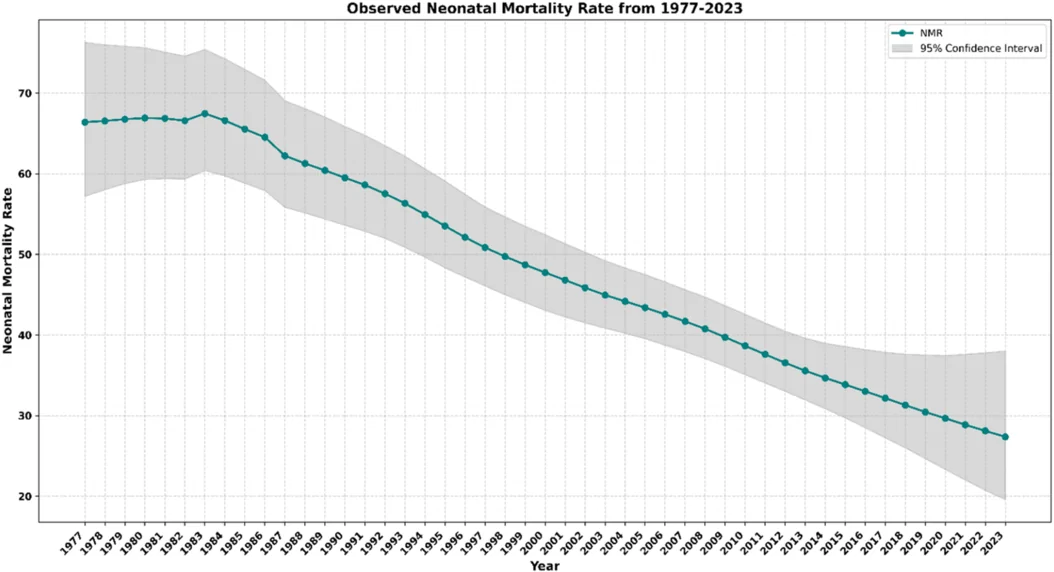
Trends in neonatal mortality rate in Ethiopia, 1977–2023.

### 3.2 ARIMA Model Identification and Diagnostic Evaluation

The testing for stationarity over time was analysed, which is essential for the ARIMA model. The rolling mean and standard deviation (Figure 3) closely follow the original data. This indicates that the mean changes over time, supporting the time series' non‐stationary nature. As the rolling standard deviation shows little variation, it suggests that when the data trend downward, the variability remains stable. Seasonality was also examined using time‐series decomposition analysis, which revealed no seasonal pattern in the data (Supplementary File). Consequently, differencing was applied to remove the underlying trend. The first‐order differencing achieves stationarity (ADF = −4.45, *p* < 0.001). Several competing ARIMA models were subsequently fitted and compared using information criteria, forecast accuracy, and residual diagnostic statistics (Supporting Information Figure S2).

**Figure 3:**
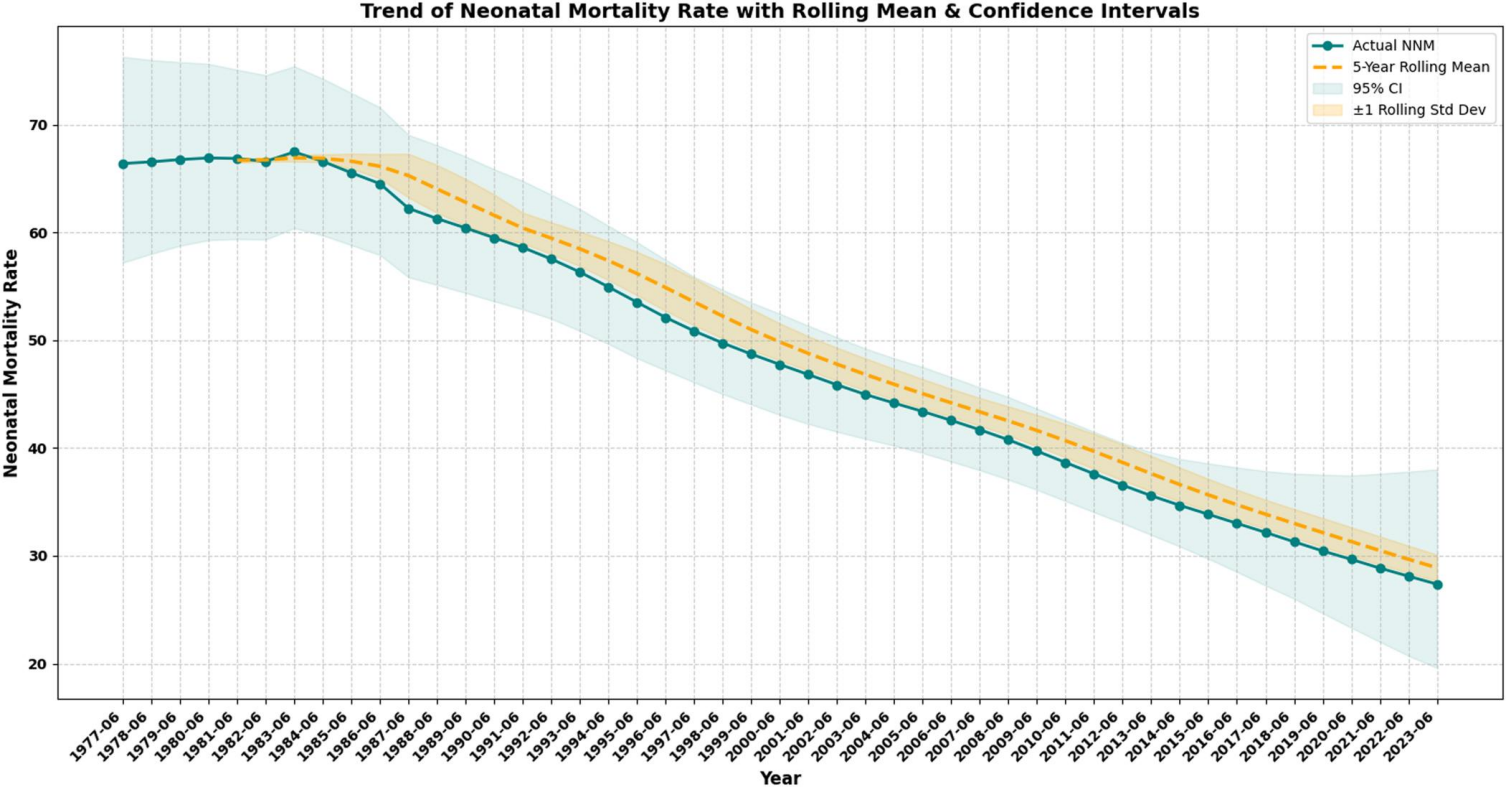
Stationarity assessment of the neonatal mortality time series data using rolling mean and rolling standard deviation.

Among the evaluated candidate models, ARIMA (2,1,2) demonstrated the best overall performance with the lowest forecasting error. Even though several competing ARIMA models produced comparable AIC, ARIMA (2,1,2) yielded the smallest forecasting errors with RMSE of 0.12, MAE of 0.10, MAPE of 0.32%, and a non‐significant Ljung–Box test with *p* = 1.00 (Supporting Information Table S1 and Supporting Information Figure S1).

### 3.3 Performance of Statistical and Deep Learning Models

Four time‐series forecasting models were developed and evaluated using the test dataset: ARIMA, single‐step LSTM, multi‐step LSTM, and the hybrid ARIMA‐LSTM model. RMSE, MAE, MAPE, sMAPE, and MASE were used as evaluation metrics.

The ARIMA (2,1,2) model had the best performance among the evaluated models, with MAE: 0.10, RMSE: 0.12, MAPE: 0.32%, sMAPE: 0.32%, and MASE:0.11. The hybrid model had comparable performance with MAE of 0.13, RMSE of 0.14, MAPE of 0.39%, sMAPE of 0.32%, and MASE of 0.13, although it did not outperform the traditional ARIMA model.

The deep learning models demonstrated substantially larger forecasting errors. The single‐step LSTM model resulted in the poorest forecasting performance, with RMSE: 8.23, MAE: 8.17, MAPE: 28.44%, sMAPE: 24.83%, and MASE: 8.74. The multi‐step LSTM improved over the conventional LSTM but remained considerably less accurate than statistical models, with RMSE: 5.31, MAE: 5.25, MAPE: 16.43%, sMAPE: 15.15%, and MASE: 5.62 (Table 1).

**Table 1:** Evaluation metrics of the forecasting models.

Model	MAE	RMSE	MAPE (%)	sMAPE (%)	MASE
ARIMA (2,1,2)	0.10	0.12	0.32	0.32	0.11
LSTM	8.17	8.23	28.44	24.83	8.74
Multistep LSTM	5.25	5.31	16.43	15.15	5.62
Hybrid	0.13	0.14	0.39	0.40	0.13

Overall, the conventional statistical forecasting models consistently outperformed the deep learning models. This superiority in ARIMA performance is likely attributable to the relatively short annual series, for which conventional statistical models are generally better suited than data‐intensive neural network architectures.

### 3.4 Forecasted Neonatal Mortality Rate (2024–2030)

According to the ARIMA (2,1,2) model, the NMR was forecast to decrease from 26.62 deaths per 1000 live births (95% PI: 25.93–27.31) in 2024 to 22.72 deaths per 1000 live births (95% PI: 17.60–27.84) by 2030 (Table 2 and Figure 4). Even though the forecasted trends suggest continued improvement in neonatal survival, the projected NMR remains substantially above the SDG target of 12 deaths per 1000 live births by 2030. The forecasting interval widened progressively over the forecast horizon, which reflects increasing uncertainty associated with long‐term projections. Nevertheless, even the lower bound of the 2030 projection interval remained above the SDG target, indicating that Ethiopia is unlikely to achieve SDG target 3.2 if current mortality trends continue.

**Table 2:** Forecasting of neonatal mortality rate in Ethiopia, 2024–2030.

Year	ARIMA (95% PI)	Single‐step LSTM (95% PI)	Multistep LSTM (95% PI)	Hybrid (95% PI)
2024	26.62 (25.93–27.31)	30.27 (28.46–32.07)	30.87 (30.04–31.71)	26.62 (26.45–26.79)
2025	25.94 (24.55–27.32)	30.33 (28.53–32.14)	29.93 (29.09–30.76)	25.94 (25.77–26.11)
2026	25.24 (23.23–27.25)	30.40 (28.60–32.21)	29.77 (28.93–30.60)	25.24 (25.07–25.41)
2027	24.59 (21.85–27.33)	30.48 (28.67–32.28)	29.44 (28.61–30.28)	24.60 (24.43–24.77)
2028	23.95 (20.46–27.43)	30.55 (28.75–32.36)	27.25 (26.42–28.09)	23.95 (23.77–24.12)
2029	23.33 (19.04–27.62)	30.63 (28.83–32.43)	26.15 (25.32–26.99)	23.34 (23.16–23.51)
2030	22.72 (17.60–27.84)	30.64 (28.84–32.45)	24.81 (23.97–25.64)	22.73 (22.56–22.91)

**Figure 4:**
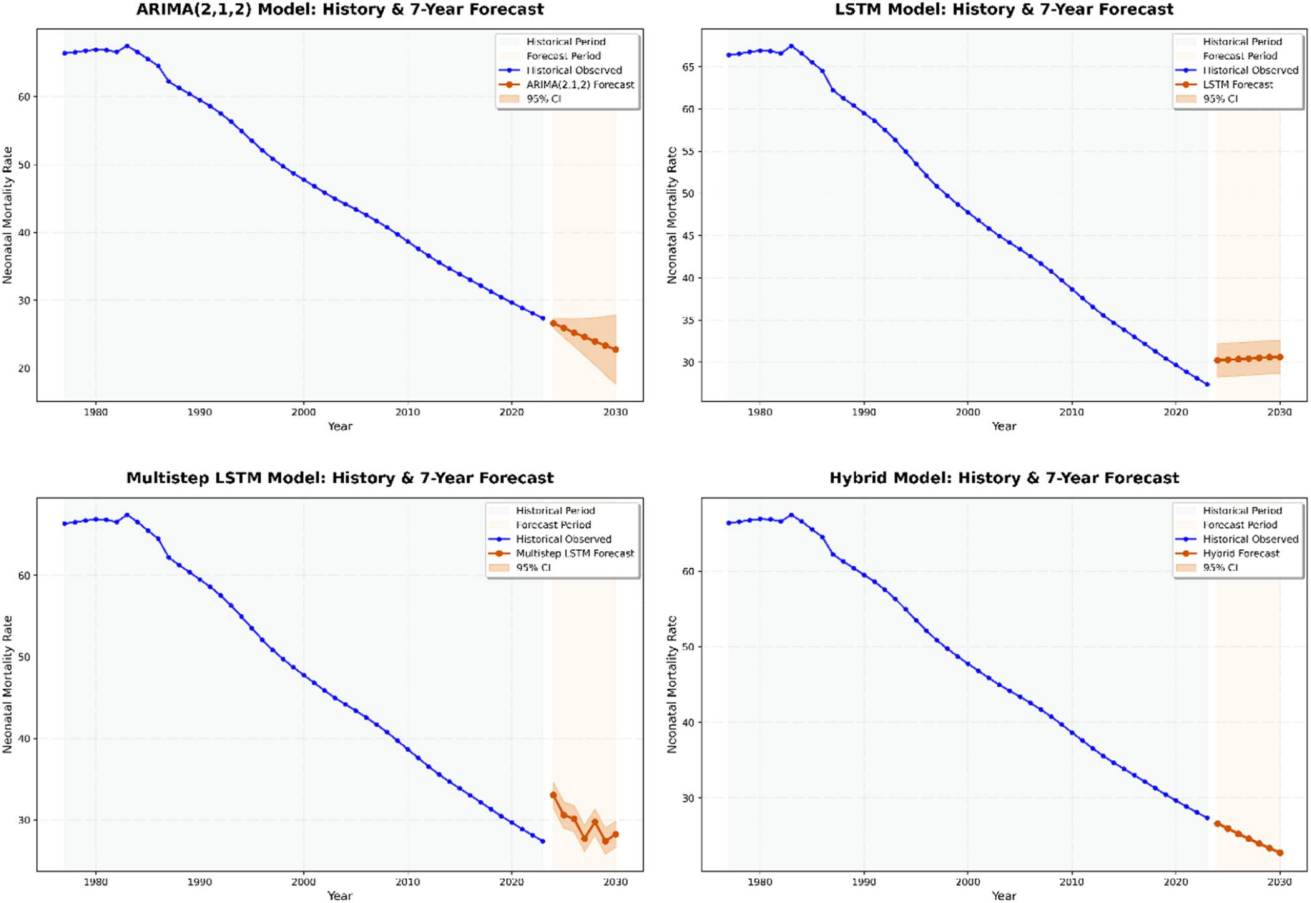
Forecasted annual neonatal mortality rate in Ethiopia, 2024–2030.

## 4 Discussion

This study reveals that the NMR in Ethiopia has declined over time, from 66 per 1000 live births in 1977 to 27 per 1000 live births in 2023, based on data from the WHO Health Inequality Data Repository. Multiple comprehensive time‐series techniques, including statistical methods such as ARIMA, deep learning methods such as single‐ and multistep LSTM, and hybrid models such as ARIMA + LSTM, were employed to forecast the NMR in Ethiopia. The performance of the models was evaluated using metrics such as MAE, RMSE, MAPE, sMAPE, and MASE. The results show that the ARIMA (2,1,2) model performed best, with MAE: 0.10, RMSE: 0.12, MAPE: 0.32%, sMAPE: 0.32%, and MASE: 0.11. This finding is consistent with [28], which highlighted that ARIMA performs well for short‐ to mid‐term predictions, small data sizes, and linear or nearly linear data, compared to LSTM, Multi‐step LSTM, and Hybrid models. Ethiopia is among six countries responsible for over half of global under‐5 mortalities. It recorded an NMR of 33 neonatal deaths per 1000 live births, which is above the sub‐Saharan African average (27 per 1000 live births) and the worldwide average of 17 per 1000 live births in 2019 [29]. The aim of SDG target 3.2 is to end preventable deaths of newborns and children under the age of 5, with the target of reducing the under‐5 mortality rate to at least 25 per 1000 live births and the NMR to at least 12 per 1000 live births in all member countries [30].

This research forecasts that by 2025, the NMR will reach 26 per 1000 live births, and by 2030, it will decline to 22.72 per 1000 live births, falling short of the SDG target. Another study with GBD concludes that Ethiopia is not on track to achieve the SDG unless it accelerates substantially [31]. This may be due to poor‐quality neonatal care in healthcare facilities, delays in care‐seeking, and barriers to access. To effectively decrease NMR in Ethiopia, it is vital to improve the quality of newborn care [32], address three delays [33], and strengthen skilled delivery [7], scale up antenatal care and postnatal care [34], target regional disparities [35], strengthen community‐based neonatal care [36], and monitor and audit neonatal deaths [37]. Moreover, the immediate optimisation of newborn and child health through the integration of advanced technologies with evidence‐based and context‐specific strategies is essential [38].

## 5 Strengths and Limitations

This study forecasts the NMR in Ethiopia using data from the WHO Health Inequality Data repository, a reliable, open‐source, and authoritative resource. The forecasting process identifies NMR patterns through assumptions and optimisation, offering a theoretical foundation for strategy development to lower NMR. Furthermore, this research makes a notable contribution to enhancing neonatal and child health in the context of the SDGs, particularly by employing artificial intelligence techniques.

However, this research has some limitations. Firstly, the WHO Health Inequality Data Repository has not been updated since 2023, and more recent estimates are unavailable. Secondly, the prediction was assessed solely through time‐series analysis; incorporating additional variables could produce more reliable results. Furthermore, no prior research in Ethiopia has used deep learning methods to benchmark outcomes against actual data, which is a limitation of our findings.

## 6 Conclusion

This study used ARIMA, LSTM, Multistep LSTM, and a hybrid model to forecast NMR in Ethiopia using 47 years of national time‐series data. Among these models, the ARIMA model performs best compared with deep learning models. These findings suggest that conventional statistical models remain well‐suited for forecasting relatively short annual health time series.

Although the NMR has declined rapidly over the past two decades, increased efforts are crucial to achieving the 2030 SDG targets. The findings also underscore the importance of accelerated implementation of effective maternal and newborn health interventions beyond the current pace of progress. The findings of this study provide valuable evidence for policymakers by demonstrating the utilisation of time‐series forecasting to monitor progress towards national and global targets. Regular updating of forecasting models with newly available data, incorporating health system, demographic, and socio‐economic predictors in future forecasting studies, may further improve prediction accuracy and support evidence‐informed planning for neonatal health in Ethiopia.

## Author Contributions

**Fentahun Bikale Kebede:** conceptualisation, investigation, funding acquisition, writing – original draft, writing – review and editing, methodology, visualisation, project administration, data curation, supervision, resources. **Selam Kifle Dergba:** conceptualisation, resources, supervision, visualisation. **Amanuel Worku:** conceptualisation, visualisation, data curation.

## Funding

The authors have nothing to report.

## Conflicts of Interest

The authors declare no conflicts of interest.

## Transparency Statement

Fentahun Bikale Kebede affirms that this manuscript is an honest, accurate, and transparent account of the study being reported; that no important aspects of the study have been omitted; and that any discrepancies from the study as planned (and, if relevant, registered) have been explained.

## Supporting information


Supporting File

## Data Availability

The data supporting the findings of this study are publicly available from the WHO Health Inequality Monitor database: https://www.who.int/data/inequality-monitor/data. Additional materials supporting the analyses are available within the article and its Supporting Materials.

## References

[1] J. E. Lawn , H. Blencowe , S. Oza , et al., “Every Newborn: Progress, Priorities, and Potential Beyond Survival,” Lancet 384, no. 9938 (July 2014): 189–205. 10.1016/S0140-6736(14)60496-7 24853593

[2] B. Terefe , F. Bikale Kebede , N. Nigussie Abrha , et al., “Multilevel Modelling of Determinants of Perinatal Mortality in East Africa: A Pooled Analysis of National Health Survey Data,” BMC Public Health [Internet] 25, no. 1 (May 2025): 2003, https://bmcpublichealth.biomedcentral.com/articles/10.1186/s12889-025-23218-w. 10.1186/s12889-025-23218-w PMC12124049 40448089

[3] V. Ridde , D. Pérez , and E. Robert , “Using Implementation Science Theories and Frameworks in Global Health,” BMJ Global Health 5, no. 4 (April 2020): e002269. 10.1136/bmjgh-2019-002269 PMC7199704 32377405

[4] United Nations Inter‐Agency Group, WHO, UNICEF, World Bank, UN DESA . United Nations Inter‐Agency Group for Child Mortality Estimation (UN IGME). Levels and Trends in Child Mortality [Internet]. 2025. Report No.: 978‐92‐806‐5636‐7, https://childmortality.org/.

[5] The World Health Organization (WHO) , World Health Statistics 2023: Monitoring Health for the SDGs, Sustainable Development Goals, 1st ed. (World Health Organization, 2023), 1.

[6] E. V. Langlois , G. Gasparri , and R. Khosla , “The Future at Risk: Tackling Newborn and Child Mortality Amidst a Global Health Crisis,” PLOS Global Public Health 5, no. 4 (April 2025): e0004519. 10.1371/journal.pgph.0004519 PMC12017561 40267077

[7] Federal Minisrty of Health and Central Statistics Agency . Ethiopia Mini Demographic Health Survey [Internet]. 2019, https://www.dhsprogram.com/.

[8] Federal Ministry of Health and Central Statistics Agency . Ethiopia Demographic Health Survey [Internet]. 2016, https://www.dhsprogram.com/.

[9] Y. Mekonnen , B. Tensou , D. S. Telake , T. Degefie , and A. Bekele , “Neonatal Mortality in Ethiopia: Trends and Determinants,” BMC Public Health 13, no. 1 (December 2013): 483, http://bmcpublichealth.biomedcentral.com/articles/10.1186/1471-2458-13-483. 10.1186/1471-2458-13-483 PMC3659057 23683315

[10] H. F. Wolde , K. A. Gonete , T. Y. Akalu , A. G. Baraki , and A. M. Lakew , “Factors Affecting Neonatal Mortality in the General Population: Evidence From the 2016 Ethiopian Demographic and Health Survey (EDHS)—Multilevel Analysis,” BMC Research Notes 12, no. 1 (December 2019): 610, https://bmcresnotes.biomedcentral.com/articles/10.1186/s13104-019-4668-3. 10.1186/s13104-019-4668-3 PMC6757386 31547855

[11] T. Tekelab , M. Akibu , N. Tagesse , T. Tilhaun , Y. Yohanes , and S. Nepal , “Neonatal Mortality in Ethiopia: A Protocol for Systematic Review and Meta‐Analysis,” Systematic Reviews 8, no. 1 (December 2019): 103, https://systematicreviewsjournal.biomedcentral.com/articles/10.1186/s13643-019-1012-x. 10.1186/s13643-019-1012-x PMC6486678 31027507

[12] M. A. Woldekidan , A. Arja , G. Worku , et al., “The Burden and Trends of Child and Maternal Malnutrition Across the Regions in Ethiopia, 1990–2019: The Global Burden of Disease Study 2019,” PLOS Global Public Health 4, no. 7 (July 2024): e0002640. 10.1371/journal.pgph.0002640 PMC11251601 39012910

[13] H. Dalili , M. Shariat , and L. Sahebi , “Time Series Analysis for Forecasting Neonatal Intensive Care Unit Census and Neonatal Mortality,” BMC Pediatrics 25, no. 1 (April 2025): 339, https://bmcpediatr.biomedcentral.com/articles/10.1186/s12887-025-05685-7. 10.1186/s12887-025-05685-7 PMC12042487 40301778

[14] A. F. M. Batista , C. S. G. Diniz , E. A. Bonilha , I. Kawachi , and A. D. P. Chiavegatto Filho , “Neonatal Mortality Prediction With Routinely Collected Data: A Machine Learning Approach,” BMC Pediatrics 21, no. 1 (December 2021): 322, https://bmcpediatr.biomedcentral.com/articles/10.1186/s12887-021-02788-9. 10.1186/s12887-021-02788-9 PMC8293479 34289819

[15] D. S. Bogale , T. M. Abuhay , and B. E. Dejene , “Predicting Perinatal Mortality Based on Maternal Health Status and Health Insurance Service Using Homogeneous Ensemble Machine Learning Methods,” BMC Medical Informatics and Decision Making 22, no. 1 (December 2022): 341, https://bmcmedinformdecismak.biomedcentral.com/articles/10.1186/s12911-022-02084-1. 10.1186/s12911-022-02084-1 PMC9795949 36577978

[16] D. A. Adeyinka and N. Muhajarine , “Time Series Prediction of Under‐Five Mortality Rates for Nigeria: Comparative Analysis of Artificial Neural Networks, Holt‐Winters Exponential Smoothing and Autoregressive Integrated Moving Average Models,” BMC Medical Research Methodology 20, no. 1 (December 2020): 292, https://bmcmedresmethodol.biomedcentral.com/articles/10.1186/s12874-020-01159-9. 10.1186/s12874-020-01159-9 PMC7712624 33267817

[17] T. Mathonsi and T. L. van Zyl , “A Statistics and Deep Learning Hybrid Method for Multivariate Time Series Forecasting and Mortality Modeling [Internet],” arXiv (2021): 1–25, https://arxiv.org/abs/2112.08618.

[18] S. Hamiane , Y. Ghanou , H. Khalifi , and M. Telmem , “Comparative Analysis of LSTM, ARIMA, and Hybrid Models for Forecasting Future GDP,” Ingénierie Des Systèmes D Information 29, no. 03 (June 2024): 853–861.

[19] G. E. P. Box , G. M. Jenkins , G. C. Reinsel , and G. M. Ljung , Time Series Analysis: Forecasting and Control, 5th ed. (John Wiley & Sons, Inc, 2016), 669.

[20] P. J. Brockwell and R. A. Davis , Introduction to Time Series and Forecasting [Internet] (Springer International Publishing, 2016). (Springer Texts in Statistics), http://link.springer.com/10.1007/978-3-319-29854-2.

[21] S. Hochreiter and J. Schmidhuber , “Long Short‐Term Memory,” Neural Computation 9, no. 8 (November 1997): 1735–1780. 10.1162/neco.1997.9.8.1735 9377276

[22] Y. Bengio , P. Simard , and P. Frasconi , “Learning Long‐Term Dependencies With Gradient Descent Is Difficult,” IEEE Transactions on Neural Networks 5, no. 2 (March 1994): 157–166. 10.1109/72.279181 18267787

[23] G. P. Zhang , “Time Series Forecasting Using a Hybrid ARIMA and Neural Network Model,” Neurocomputing 50 (January 2003): 159–175.

[24] S. Siami‐Namini , N. Tavakoli , and A. Siami Namin , “A Comparison of ARIMA and LSTM in Forecasting Time Series.” 2018 17th IEEE International Conference on Machine Learning and Applications (ICMLA) [Internet] (IEEE, 2018), 1394–1401, https://ieeexplore.ieee.org/document/8614252/.

[25] K. Kirkby , N. Bergen , A. Baptista , A. Schlotheuber , and A. R. Hosseinpoor , “Data Resource Profile: World Health Organization Health Inequality Data Repository,” International Journal of Epidemiology 52, no. 5 (October 2023): e253–e262. 10.1093/ije/dyad078 PMC10555845 37322565

[26] H. M. Luk , E. Allanson , W. K. Ming , and W. C. Leung , “Improving Diagnostic Classification of Stillbirths and Neonatal Deaths Using ICD‐PM (International Classification of Diseases for Perinatal Mortality) Codes: Validation Study,” JMIR Medical Informatics 8, no. 8 (August 2020): e20071. 10.2196/20071 PMC7432142 32744510

[27] F. A. Gers , J. Schmidhuber , and F. Cummins , “Learning to Forget: Continual Prediction With LSTM,” Neural Computation 12, no. 10 (October 2000): 2451–2471. 10.1162/089976600300015015 11032042

[28] Z. K. Adama , F. O. Mettle , B. M. Baiden , and N. K. Bii , “Forecasting Progress: Analyzing the Trajectory of Under‐Five Child Mortality for Ghana, Niger, Nigeria, and Sierra Leone Towards SDG3 Using ARIMA Time Series Model,” BMC Public Health [Internet] 25, no. 1 (May 2025): 1607, https://bmcpublichealth.biomedcentral.com/articles/10.1186/s12889-025-22869-z. 10.1186/s12889-025-22869-z PMC12044808 40312307

[29] G. A. Tessema , T. M. Berheto , G. Pereira , A. Misganaw , and Y. Kinfu , GBD 2019 Ethiopia Child Mortality Collaborators , “National and Subnational Burden of Under‐5, Infant, and Neonatal Mortality in Ethiopia, 1990–2019: Findings From the Global Burden of Disease Study 2019,” PLOS Global Public Health 3, no. 6 (June 2023): e0001471. 10.1371/journal.pgph.0001471 PMC10284418 37343009

[30] United Nations , “Transforming Our World: The 2030 Agenda for Sustainable Development,” [Internet], (2015), https://sdgs.un.org/goals/goal3.

[31] A. Misganaw , M. Naghavi , A. Walker , et al., “Progress in Health Among Regions of Ethiopia, 1990–2019: A Subnational Country Analysis for the Global Burden of Disease Study 2019,” Lancet 399, no. 10332 (April 2022): 1322–1335, 10.1016/S0140-6736(21)02868-3. PMC8987934 35294898

[32] B. Tekeba , T. T. Tamir , B. S. Workneh , et al., “Early Neonatal Mortality and Determinants in Ethiopia: Multilevel Analysis of Ethiopian Demographic and Health Survey, 2019,” BMC Pediatrics [Internet] 24, no. 1 (August 2024): 558, https://bmcpediatr.biomedcentral.com/articles/10.1186/s12887-024-05027-z. 10.1186/s12887-024-05027-z PMC11363417 39215240

[33] E. Garcia Gomez , K. A. Igunza , Z. J. Madewell , et al., “Identifying Delays in Healthcare Seeking and Provision: The Three Delays‐in‐Healthcare and Mortality Among Infants and Children Aged 1–59 Months,” PLOS Global Public Health 4, no. 2 (February 2024): e0002494. 10.1371/journal.pgph.0002494 PMC10852234 38329969

[34] G. A. Fekadu , G. M. Kassa , A. K. Berhe , A. A. Muche , and N. A. Katiso , “The Effect of Antenatal Care on Use of Institutional Delivery Service and Postnatal Care in Ethiopia: A Systematic Review and Meta‐Analysis,” BMC Health Services Research [Internet] 18, no. 1 (December 2018): 577, https://bmchealthservres.biomedcentral.com/articles/10.1186/s12913-018-3370-9. 10.1186/s12913-018-3370-9 PMC6056996 30041655

[35] G. Shibre , D. Idriss‐Wheeler , and S. Yaya , “Inequalities and Trends in Neonatal Mortality Rate (NMR) in Ethiopia: Evidence From the Ethiopia Demographic and Health Surveys, 2000–2016,” PLoS One 15, no. 6 (June 2020): e0234483. 10.1371/journal.pone.0234483 PMC7286487 32520940

[36] B. I. Avan , D. Berhanu , Y. Mekonnen , et al., “Embedding Community‐Based Newborn Care in the Ethiopian Health System: Lessons From a 4‐Year Programme Evaluation,” supplement, Health Policy and Planning 36, no. Suppl_1 (November 2021): i22–i32. 10.1093/heapol/czab085 PMC8633669 34849897

[37] K. K. Cyprien , D. Augustine , D. Isabelle , et al., “Audit of Neonatal Deaths According to the Three Delays Model in a Referral Hospital,” Open Journal of Pediatrics 15, no. 02 (2025): 189–197.

[38] P. Duran , J. A. Sommer , P. Otero , et al., “Information and Communication Technologies in Neonatal Health,” Revista Panamericana de Salud Pública 44 (November 2020): 1. 10.26633/RPSP.2020.123 PMC7655061 33196698

