# Supplementary material for: Forecasting Ethiopia's Neonatal Mortality Rate Toward the Sustainable Development Goal Target 3.2 Using Statistical and Deep Learning Models: A Time‐Series Forecasting Study
Source: Health Sci Rep. 2026 Oct 21;9(10):e73402. doi: 10.1002/hsr2.73402 (PMC13685530; doi:10.1002/hsr2.73402)
Supplement: Supplementary file 1 [file HSR2-9-e73402-s001.docx]

**Supplementary Table S1:** *Comparison of candidate ARIMA models based on forecasting performance, information criteria, and residual diagnostics.*

| **Model** | **RMSE** | **MAE** | **MAPE (%)** | **AIC** | **BIC** | **LB_p-value** |
| --- | --- | --- | --- | --- | --- | --- |
| ARIMA (2, 1, 2) | 0.12 | 0.10 | 0.32 | 49.08 | 57.00 | 1.0 |
| ARIMA (1, 1, 1) | 0.14 | 0.12 | 0.38 | 49.06 | 53.82 | 1.0 |
| ARIMA (3, 1, 0) | 0.15 | 0.13 | 0.42 | 50.07 | 56.41 | 1.0 |
| ARIMA (3, 1, 1) | 0.16 | 0.14 | 0.46 | 44.65 | 52.56 | 1.0 |
| ARIMA (2, 1, 1) | 0.23 | 0.18 | 0.60 | 50.74 | 57.08 | 1.0 |
| ARIMA (3, 1, 2) | 0.23 | 0.19 | 0.64 | 46.26 | 55.76 | 1.0 |
| ARIMA (2, 1, 0) | 0.48 | 0.35 | 1.21 | 49.33 | 54.08 | 1.0 |
| ARIMA (1, 1, 3) | 0.55 | 0.45 | 1.53 | 48.05 | 55.96 | 1.0 |
| ARIMA (1, 1, 2) | 0.73 | 0.53 | 1.83 | 50.48 | 56.82 | 1.0 |
| ARIMA (2, 1, 3) | 1.44 | 1.08 | 3.75 | 49.12 | 58.62 | 1.0 |
| ARIMA (1, 1, 0) | 1.64 | 1.28 | 4.41 | 53.07 | 56.24 | 1.0 |
| ARIMA (3, 1, 3) | 2.09 | 1.68 | 5.76 | 43.89 | 54.97 | 1.0 |
| ARIMA (0, 1, 3) | 4.13 | 3.47 | 11.82 | 50.22 | 56.56 | 1.0 |
| ARIMA (0, 1, 2) | 4.56 | 3.94 | 13.36 | 72.61 | 77.36 | 1.0 |
| ARIMA (0, 1, 1) | 4.72 | 4.09 | 13.87 | 76.88 | 80.05 | 1.0 |
| ARIMA (0, 1, 0) | 5.19 | 4.62 | 15.61 | 105.42 | 107.00 | 1.0 |

***Supplementary Figure 1:*** *Summary output of the fitted ARIMA (2,1,2) model for neonatal mortality rate forecasting in Ethiopia*

***Supplementary Figure 2:*** *Diagnostic evaluation of residual assumptions for the forecasting model:* ***(a)*** *standardised residuals,* ***(b)*** *residual distribution and normality assessment, and* ***(c)*** *autocorrelation function analysis of residuals.*
